# Diagnosis System for Hepatocellular Carcinoma Based on Fractal Dimension of Morphometric Elements Integrated in an Artificial Neural Network

**DOI:** 10.1155/2014/239706

**Published:** 2014-06-16

**Authors:** Dan Ionuț Gheonea, Costin Teodor Streba, Cristin Constantin Vere, Mircea Șerbănescu, Daniel Pirici, Maria Comănescu, Letiția Adela Maria Streba, Marius Eugen Ciurea, Stelian Mogoantă, Ion Rogoveanu

**Affiliations:** ^1^Research Center of Gastroenterology and Hepatology, University of Medicine and Pharmacy of Craiova, Petru Rares Street, No. 2, 200349 Craiova, Romania; ^2^Department of Medical Informatics, University of Medicine and Pharmacy of Craiova, Petru Rares Street, No. 2, 200349 Craiova, Romania; ^3^Department of Histology, University of Medicine and Pharmacy of Craiova, Petru Rares Street, No. 2, 200349 Craiova, Romania; ^4^Department of Pathology, University of Medicine and Pharmacy “Carol Davilla,” Bucharest, Bulevardul Eroii Sanitari 8, 050474 București, Romania; ^5^2nd Medical Department, University of Medicine and Pharmacy of Craiova, Petru Rares Street, No. 2, 200349 Craiova, Romania; ^6^Department of Surgery, University of Medicine and Pharmacy of Craiova, Petru Rares Street, No. 2, 200349 Craiova, Romania

## Abstract

*Background and Aims*. Hepatocellular carcinoma (HCC) remains a leading cause of death by cancer worldwide. Computerized diagnosis systems relying on novel imaging markers gained significant importance in recent years. Our aim was to integrate a novel morphometric measurement—the fractal dimension (FD)—into an artificial neural network (ANN) designed to diagnose HCC.* Material and Methods.* The study included 21 HCC and 28 liver metastases (LM) patients scheduled for surgery. We performed hematoxylin staining for cell nuclei and CD31/34 immunostaining for vascular elements. We captured digital images and used an in-house application to segment elements of interest; FDs were calculated and fed to an ANN which classified them as malignant or benign, further identifying HCC and LM cases.* Results.* User intervention corrected segmentation errors and fractal dimensions were calculated. ANNs correctly classified 947/1050 HCC images (90.2%), 1021/1050 normal tissue images (97.23%), 1215/1400 LM (86.78%), and 1372/1400 normal tissues (98%). We obtained excellent interobserver agreement between human operators and the system.* Conclusion*. We successfully implemented FD as a morphometric marker in a decision system, an ensemble of ANNs designed to differentiate histological images of normal parenchyma from malignancy and classify HCCs and LMs.

## 1. Introduction

Hepatocellular carcinoma (HCC) represents a major health concern as it represents the third cause of cancer-related mortality [1, 2] and fifth in incidence [1–5], being at the same time the second most prevalent liver tumor (after liver metastases) and first hepatic primary malignancy [2]. Curative treatment is reserved for early stages of the disease; tumor size and number as well as the state of previous liver disease play an essential role [6–8]. Therefore, early diagnosis and the requirement for precise identification are essential for improving the management of these patients. The latest guidelines underline the need for new biomarkers designed to identify responders to therapy and for trial enrichment [8].

As the primary diagnosis methods for HCC rely on contrast-enhanced arterial uptake imaging, liver pathology is usually reserved to tumors of undetermined origin or too small to be properly investigated noninvasively, or for the evaluation of treatment in clinical or experimental trials [7, 8]. Hence, the clinician needs accurate and rapid diagnosis on small pathology samples.

The use of computerized aided methods for histological evaluation of tissue samples has been present in usual practice for several decades; however, great improvements were obtained in recent years, with the rapid evolution of computational methods and the increased complexity of algorithms. Fractal image analysis with the determination of fractal dimension (FD) emerged as useful tools for classification of natural shapes that do not follow normal geometrical conformations [9, 10]. This method is nowadays used in pathology [11–16] as well as in other medical imaging fields, providing precise quantification for various elements. The spatial distribution profile in relation to the gauge of a given space represents the basis for most methods of calculating the fractal dimension, models based on the work of Hausdorff and Kolmogorov being the most suitable for quantitative appreciation [17–19].

Computer-aided diagnosis (CAD) systems are independent decision systems employed in medical management of several pathologies, with an emphasis on various malignancies [20–24]. From the multitude of currently available artificial intelligence systems, the most useful tools have been proven to be based on machine learning (ML), especially artificial neural networks (ANN). Found in various iterations, ANN systems mimic the architectonics of the human brain in order to solve classification problems, thus making excellent decision-making systems in medical diagnostics and prognosis [22–24].

In this paper, we present a novel application of fractal image analysis with FD calculation and integration in an ANN model for classifying liver tumors and especially HCC diagnosis.

## 2. Materials and Methods

### 2.1. Patient Selection

The study was conducted in accordance with the Declaration of Helsinki. Patients were not subjected to any investigation or operatory maneuver other than those appropriate for their condition. Informed consent for all procedures was obtained as standard procedure required and supplemental written acknowledgement on the inclusion in the study was given prior to manipulation and interpretation of the histological fragments. The Ethical Committee of the University of Medicine and Pharmacy of Craiova as well as that of the University Hospital expressed their consent to the study.

We prospectively included between January 2010 and December 2012 a total of 49 patients scheduled for surgery—21 with HCC and 28 with liver metastases (LM). Interventions were performed within the Department of Surgery, University Hospital of Craiova. Tumors were removed along with healthy tissue during lobectomies following usual protocols, and we collected fresh tissue samples for histological processing and analysis.

Patients were selected from those diagnosed with liver tumors scheduled for surgery with the intent of performing liver resection. For HCC this implied patients with Stage 0 of disease, according to the Barcelona criteria [6, 8]. After the intervention, we surveyed the patients for a minimum period of six months to make sure the initial diagnosis was correct and to evaluate their postoperative condition.

### 2.2. Pathology Specimens and Expert Interpretation

Hematoxylin staining was used for nuclear chromatin disposition and immunohistochemical staining with CD34/CD31 antibodies was used for assessing vascular patterns. Both techniques have been previously described in detail by our group [25]. Briefly, for immunostaining we used overnight incubation with an anti-CD34 and anti-CD31 antibody cocktail (Clones QBEnd-10, Dako, and, JC70A, 1 : 100 dilution, resp.) and then used a polymer-HRP system for amplification (Dako, Denmark). Detection was performed with 3′3′diaminobenzidine (Dako, Denmark) and counterstained with hematoxylin for marking the nuclei.

A total of ten slides were prepared for each case, five with tumoral parenchyma and five with normal surrounding tissue. We then proceeded to record 100 consecutive images (10 per each slide) and digitally stored them for each patient. We used a Nikon Eclipse 90i microscope (Apidrag, Bucharest, Romania) with apochromatic 40x and 60x objectives for imaging, coupled to a dedicated 5-megapixel CCD camera for recording the uncompressed images.

Two pathologist experts in diagnosing HCC and LM, blinded to the initial diagnosis, gave their interpretation on each image (DP and MC). Their assessment was tested with the agreement coefficient by using the weighted* Kappa* test. The strength of the agreement as expressed with the Kappa coefficient [26] was quantified as negligible (values between 0.00 and 0.20), slight (0.21–0.40), moderate (0.41–0.60), great (0.61–0.80), and excellent (0.81–1.00).

### 2.3. Image Processing and Calculation of FDs

**Figure 1:**
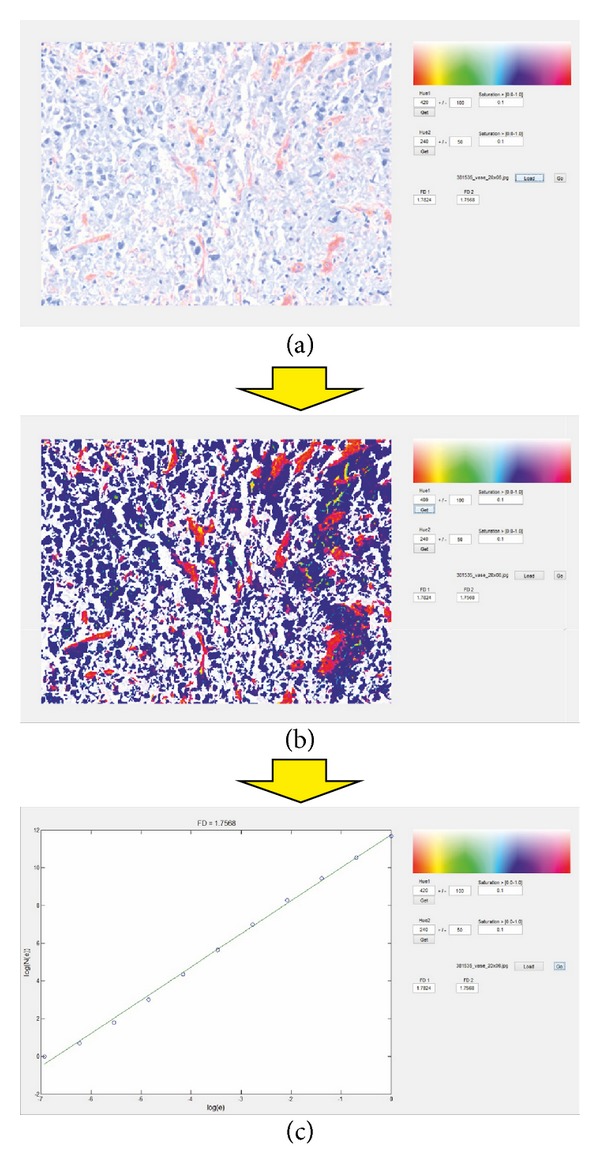
(a)–(c) An overview of the interface designed for selecting and calculating FDs. (a) An individual image is loaded; then values for the reference hues and standard deviations as well as the desired saturations can be either typed in the designated box or manually selected with the mouse cursor on the image representation on the left. (b) The operator receives a visual confirmation of the selection and can further adjust the parameters in order to make it more accurate. (c) The FD is calculated and a graphical representation of the log-log function is presented to the user. After the cycle is completed, the user can batch-process entire series of images. Values for FDs are automatically saved as lists of comma-separated values and are fed to the ANN system.

**Figure 2:**
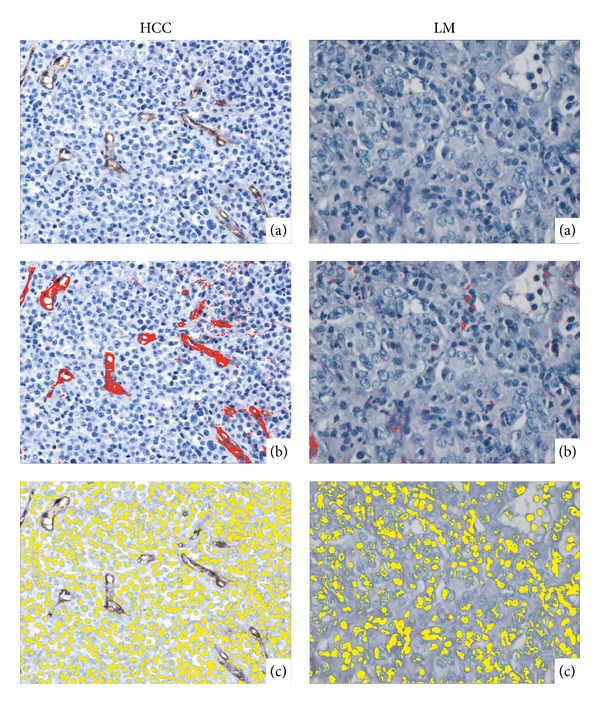
(a)–(c) The process of selecting vessels and nuclei for calculating FDs. (a) The initial pathology images. The pathologist can observe the large pleomorphic nuclei and the enlarged nucleus/cytoplasm ratio as well as the prominent nucleoli. Also, after immunohistochemical staining for newly formed blood vessels, the relative paucity in the case of LM as opposed to HCC can be observed. (b) The vessels are selected automatically by the software. (c) The same process is applied on the predefined color channels for cellular nuclei.

Uncompressed images in Bitmap format were processed in a custom-created computer program. The software was created as an application in MATLAB (MathWorks, USA). The software interface is presented in Figure 1. The process through which blood vessels and cellular nuclei were selected is illustrated in Figure 2.

The image color space was translated from red-green-blue (RGB) signature to a hue-saturation-value (HSV) defined color space which allowed segmenting each image and extracting the nuclear signatures and vascular axels. The software threshold eliminated any element under 10 pixels, considered artifacts. This step also allowed us to ignore incomplete nuclei which did not fit entirely in the imaging field.

In order to make it even easier to segment the nuclei or vascular elements, the “value” parameter was set constant; thus, the whole color space became two-dimensional. By using a color threshold and selecting the “blue” or the “brown” pixels from the images we were able to clearly determine the nuclei/vascular elements from the background and automate the selection.

By using the fractal box-counting algorithm, FDs were obtained as the regression slope of the regression line for the log-log plot of the scanning box size and the count from a box-counting scan.

A fractal dimension is a synthetic index for characterizing fractal patterns or sets by quantifying their complexity as a ratio of the change in detail to the change in scale. Basically, in our approach, this is a non-Euclidian morphological parameter that aims to quantify the roughness or the irregularity of the perimeter line of the nuclei or of the vessels' outlines.

Starting from the general formula of the FD, consider *e* as the box length (scale), *N*(*e*) as the number of boxes required to cover the structures (detail), FD as the fractal dimension, and *C* as a constant number:

(1) $$\begin{array}{c} N\left(e\right)=C*e^{\text{DF}}. \end{array}$$

We obtained the numerical approximation of the FD:

(2) $$\begin{array}{c} \text{FD}=\text{slope}\left(\frac{\text{Log}\left(N\left(e\right)\right)}{\text{Log}\left(e\right)}\right). \end{array}$$

The nuclear fractal dimension (FD) on each image was estimated by using an in-house implementation of the box-counting algorithm using MATLAB. The algorithm returned the FD of a binary image object using the polyfit MATLAB standard function for obtaining the slope and was designed as follows.

1. Pad the image with background pixels so that its dimensions are a power of 2 (0 is background).
2. Set the box size “*e*” to the size of the image.
3. Compute *N*(*e*), which corresponds to the number of boxes of size “*e*” which contains at least one object pixel.
4. If *e* > 1 then *e* = *e*/2 and repeat step (3).
5. Compute the points log⁡(*N*(*e*)) × log⁡(1/*e*).
6. Use the least squares method to fit a line to the points.
7. The returned FD (Hausdorff) is the slope of the line.

We registered and automatically analyzed 100 images per patient (10 images for each slide, 10 slides equally divided between tumor and normal parenchyma), totaling at 4900 images for all cases. This high number ensured a large enough sample for the ANN system described below.

### 2.4. Computerized Diagnostic System Based on Artificial Neural Networks

**Figure 3:**
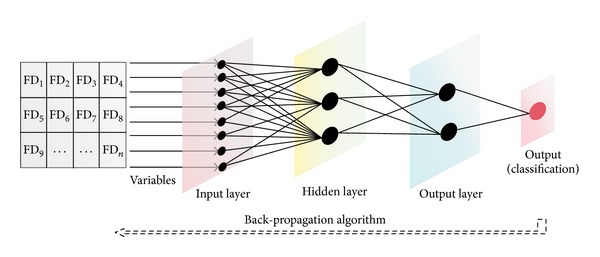
Graphical representation of an ANN. The FDs are imputed to corresponding neurons in the first layer of the ANN, which in turn send the data to all neurons of the hidden layer. The neurons in this intermediate layer establish an importance value for the output layer, which presents the user with a result, classifying the image into one category.

The resulting FDs were automatically fed by the software application to a double-layer feed-forward ANN designed to classify images as malignant or benign; furthermore, a second ANN collected all mean FDs of malignant images from each case and determined if the tumor was more likely to be HCC or LM. Both the ANN models were developed in MATLAB and fully integrated within the graphical interface. As we established from previous work, optimal ANN layout for classification tasks in image processing is usually the simplest. Thus, we chose the network architecture to contain only one hidden layer, with an input layer and one layer dedicated for the output (Figure 3).

Briefly, ANNs are made up of multiple interconnected units called “neurons,” each containing a transfer function. They are organized in “layers” which usually perform a function—most ANN models contain, for instance, one input layer (for receiving the parameters) and one output layer for giving results, based on calculations made in intermediate layers by interconnected neurons. Neurons are connected by “synapses” and the ones in the intermediate layers attribute “weights” to each variable, based on the strength of the connection. If a connection is used multiple times to reach a solution, that parameter gains importance towards a decision, thus establishing a hierarchy within the system [27–29].

In our model, neurons in the hidden layer of the first ANN associated transfer and processing functions for each FD of all elements in a given image. The sum of products between synaptic weights and neuron values classified them as benign and malignant (i.e., identifying if it is an image of normal parenchyma or from the tumor area). Similarly, the second ANN received the mean FDs per malignant image for each set corresponding to a patient and attributed weights to each value. By summing them this ANN reached a conclusion of either HCC or LM. The suggestions given by the networks also received a probability score—the percentage from the ideal score for the ideal value.

For both ANN models, the sets of FDs (for elements in an image and for each patient, resp.) were randomly divided into training, validation, and testing sets, respectively (50% training, 25% validation, and 25% testing). In short, during the training phase the system learned how to classify an image by comparing the values obtained in the evaluation with the correct diagnosis, therefore establishing the weights of each synapse. We used a back-propagation algorithm and 10-fold cross validation which we previously used [27–30] in order to minimize overfitting (ultraclassify based on rigid rules). We were able to change the learning rate and determine the number of epochs (iterations needed for completion of the training phase) and perform adjustments in real time.

**Figure 4:**
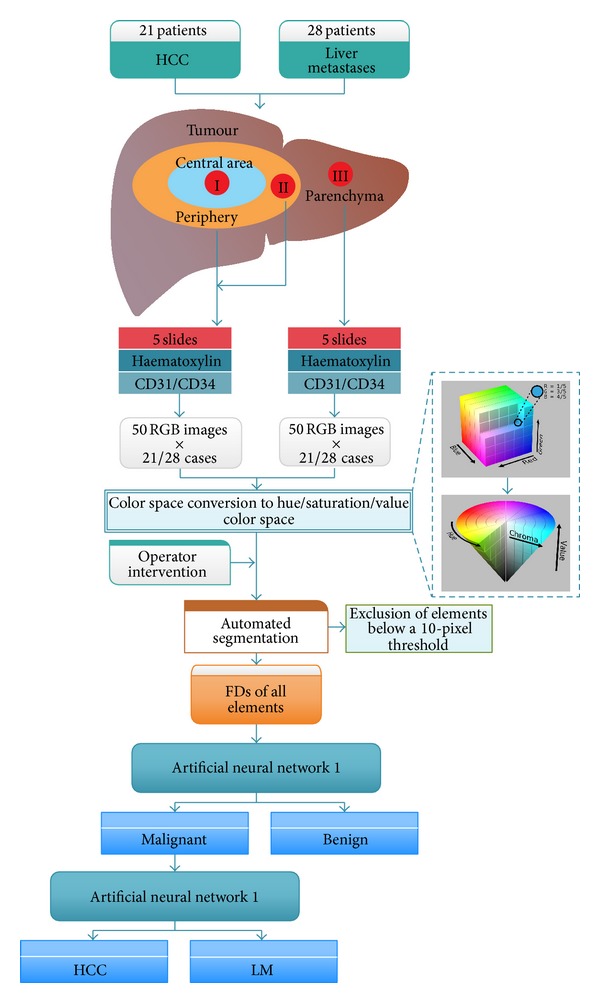
Overview of the study protocol. Tissue samples from the liver resection pieces of 49 patients with either HCC (21) or LM (28) undergo hematoxylin staining and CD31/34 immunohistochemistry. RGB images are converted in the HSV space and elements are semiautomatically segmented with the calculation of FDs for each element, either cell nuclei or vascular axels. Elements below a 10-pixel threshold are automatically excluded, and the remaining data is fed to a first ANN which classifies the image as either malignant or benign. All malignant images are further classified by a 2nd ANN into either HCC or LM. (The RGB and HSV images provided as examples are reproduced from http://commons.wikimedia.org/wiki/User:SharkD and were originally licensed under the Creative Commons Attribution-Share Alike 3.0 Unported license.)

A workflow of the whole study protocol can be observed in Figure 4.

## 3. Results

### 3.1. Characteristics of the Patient Lot

We included a total of 49 patients (37 men) who met the inclusion criteria and were available for at least six months of followup. Their characteristics are summarized in Table 1. No patient died or dropped out during the follow-up period. We could observe that tumors were more prevalent in men (37 men versus 12 women), with a 4.25 : 1 male : female ratio for HCC and 2.5 : 1 ratio for LM. All but one of the HCC patients had a history of chronic viral hepatitis infection, either B (12 cases, 9 with cirrhosis) or C (6 cases, 3 with cirrhosis); we also found two cases with cirrhosis of mixed B and C viral etiology. All LM cases had other prediagnosed tumors (14 colon adenocarcinomas found during colonoscopy, 12 lung cancers confirmed by chest radiograph and CT scan, and two gastric cancers confirmed on endoscopy) for which they underwent curative treatment (data not shown). One LM patient had a history of chronic viral C hepatitis. We also acknowledged the importance of both alcohol intake and smoking as a risk for developing cancer, with both habits being highly prevalent in our patient lots.

All HCCs were single tumors with diameters below 2 cm, making them ideal candidates for liver resection. For LM, 5 cases presented multiple tumors; the median diameters were approximately three times higher than those of HCCs.

### 3.2. Human Histological Interpretation

The two pathologists randomly reviewed the images recorded from slides and gave their expert opinion based on the overall appearance of the image. The Kappa coefficient was calculated and the agreement between the two observers was found to be excellent (Kappa of 0.998; standard error of Kappa = 0.001; 95% confidence interval = 0.996–0.999).

The first pathologist correctly identified 99.6% of all HCC images while the second pathologist correctly identified 99.4% of all HCC images (Table 2); four images were misinterpreted by both as being LM. In the case of LM, the first pathologist identified 98.4% of the images and the second pathologist identified 98.8% of the images (Table 3); 17 images were misinterpreted by both as being HCC. No normal parenchyma image was misinterpreted by any of the two pathologists, and no image from any of the tumors was interpreted as normal. When they reviewed all images from each case, both pathologists gave the correct diagnosis on a per case basis.

### 3.3. Fractal Analysis of Histological Images

The interface allowed us to select the hue interval and the saturation level manually and set the reference values for all images. This was possible for two independent elements, in our case for cellular nuclei and vascular vessels (see Figures 1(a) and 1(b)). Visual inspection of a given image was possible; however, FDs were batch-calculated by the software (Figure 1(c)). Overall, the automated segmenting algorithm correctly selected 92% of the HCC image sets (1932/2100) and 90% of the metastases sets (2520/2800). Manual corrections were applied to the other images in order to provide an accurate FD calculation (i.e., not to over- or underselect a certain element, either nuclei or vascular vessels). We thus obtained the two sets of variables to be fed into the first ANN system—the FDs for nuclei and for vascular elements (see Table 4 for an overview of the data).

Once the ANN decided an image represented a malignant area, the software calculated two median FDs per image and further fed the data on a per patient basis to the second ANN system, in order to evaluate whether it is a case of HCC or of LM (see Figure 3 for details on the working protocol).

### 3.4. The ANN Decision System

The first ANN system successfully identified all 2450 images obtained from the two tumor types (Table 5). However, the system misinterpreted 57 (2.32%) images of healthy parenchyma as being malignant. The overall sensitivity and specificity of the first ANN were 100% and 97.6%, respectively.

The second ANN system thus received 2507 images as malignant (including the 57 misinterpreted images from the first ANN analysis). Its sensitivity was 90.19% and specificity was 86.78%. It correctly classified 947/1050 (90.2%) and 1215/1400 metastases (86.78%) and classified the wrongly included parenchyma images into HCC (27 images) and LM (30 images).

We then proceeded to calculate the Kappa level of agreement between the CAD system and the two human operators, obtaining excellent agreement in both comparisons. The results are presented in Table 6.

## 4. Discussions

We present here what we believe is the first automated system to integrate fractal image analysis of liver tumors and parenchyma into a computer-aided diagnostic system, by providing fractal dimensions to a combined system of single-layer feed-forward artificial neural networks that can classify histology images into liver primary or secondary and recognize normal parenchyma.

We have previously reported in a pilot study [25] the first results of using FD in discriminating between HCC and various cases of LM; our previous results showed good discriminating capabilities of this morphometric parameter on a small number of cases with a large number of extracted images. We now established on this extended lot of prospectively selected patients that indeed FD can be used to discriminate between malignant and benign histology images and more specifically can differentiate HCC from LM.

Fractal analysis relies on the morphological complexity and the intrinsic self-similarity that most natural shapes occurring in nature possess. Calculation of FD is performed by quantifying the ability of an item to fill the space it resides in. For bidimensional structures that are represented in the plane of a digitized image, for instance, the FD can only be between 1 (corresponding to a straight line) and 2 (a filled circle that matches the density of the space). Usage of FD has proved to be beneficial in pathology, as various structures could be evaluated in this manner, especially in neural structures, tumor angiogenesis, or fibrotic processes within the liver [31–35].

The RGB color model is good for presenting images on a computer screen to a human operator, as it mimics the human eye model. The problem with its Cartesian representation (a cube with each axis representing a base color) is that it is nonintuitive in the matter of the “next” or closest color when trying true images color comparisons. Moreover, we used real images from histological stained tissue in which we were interested in brown (CD31/34 immunolabeling for vessels) and blue (hematoxylin for nuclei) colors. It was thus possible to have similar values of both colors in the same pixel and therefore we could not decide if it is part of the element of interest or of the surrounding elements. The HSV is one of the most common cylindrical-coordinate representations of points in an RGB color model. The representation rearranges the geometry of RGB in an attempt to be more intuitive and perceptually relevant than the Cartesian representation.

Delides et al. [16] used FD as a prognostic factor for laryngeal carcinoma, while Goutzanis and his team proved that increased FD for cellular elements is inversely correlated with survival in oral cancer. The results presented in our study can be successfully applied when analyzing response to chemotherapy. It can evaluate the posttreatment state of newly formed blood vessels after Sorafenib usage and can possibly stratify patients according to response rates.

A very recent study concluded that if a resected liver tumor contains poorly differentiated components, it is safe to assume poor prognosis and high recurrence rates [36]. The system described here can find its utility in this field, as FDs are sensitive morphometric tools for assessing cellular and vascular features. Other previous studies also theorized on the usage of FD in cancer prognosis [34, 35]; our system integrates ANNs into the diagnosis and therefore can increase the specificity and sensitivity of such a method.

Our system relied on a perceptron feed-forward hidden-layer ANN and used back-propagation algorithms. This setup was proven to be the better choice when designing medical diagnosis tools; its simple architecture is best suited to avoid overfitting and is at the same time one of the fastest available [20, 27]. The use of automated image interpretation tools in medicine greatly depends on the quality of the data received by the system, and machine-based learning systems can increase the accuracy of any image analysis tool that is based on quantitative assessment of feature elements [37–40]. The effectiveness of this layout was proven by excellent training and validation times, with few cycles being sufficient for optimal results. The testing phase maintained the fast rates already shown in the previous phases. We calculated the Kappa coefficient in order to determine the interobserver agreement between the ANN system and human operators and found excellent correlation between their interpretations.

The system can therefore be integrated in training applications for medical practitioners or can serve as an independent assessor for aiding pathologists in presenting a diagnosis. It will be available on the World Wide Web as a free online tool and can be accessed at the address hepfracnet.umfcv.ro. Current imaging analysis methods are heavily dependent on the experience of the pathologist, and they rely on subjective interpretations; introducing a parameter that is size irrelevant such as FD in conjunction with a learning system can prove to be extremely beneficial for improving both the time needed for a diagnosis and medical decisions. Previous studies [22–24] showed that both tumor grade and vascular invasion can be predicted by the use of ANNs; furthermore, clinical decision-making can benefit from the use of computer-aided diagnostic systems.

Our study suffers from some limitations; HCC patients that qualify for liver resection are usually selected from those with an early diagnostic and are therefore somewhat less frequent. Therefore, we could not include a large number of patients and also did not try to stratify the lot based on histological grading. We believe that in such low numbers the system can be able to differentiate different stages of HCC; however, these results may not be reproducible in larger cohorts. Our pilot statistical study on the usage of FDs for HCC diagnosis [25] showed promising results in terms of identifying the type of LM; however, as we already predicted, these results could not be reproduced in this larger-scale design. The study may benefit from an increased number of heterogeneous images from more HCC cases, as the nature of ANNs is to evolve with an increased training dataset. Also, experimenting with other architectures or types of machine learning techniques may provide improved results. The use of other patient data (from both patient history and blood tests, for instance) as well as imaging parameters can greatly improve the accuracy of a CAD system in diagnosing liver focal lesions. In this manner, as some of our previous researches suggest, metastases can be further classified according to origin and a multitude of other liver lesions may be well diagnosed [27–29].

In conclusion, we successfully proved that nuclear and vascular FDs calculated from histological images are good quantifiers for morphological aspects of liver parenchyma and can therefore fit perfectly as input variables in a perceptron feed-forward hidden-layer ANN system. Our implementation could distinguish between malignant and benign histological images and further classify malignant images into either HCC or LM, thus distinguishing between primary and metastatic tumors within the liver. This system may have excellent applications in telemedicine, medical training, or time-efficient diagnostic of HCC cases and can positively influence response to both surgical and drug-based treatments.

## Figures and Tables

**Table 1:** Characteristics of the patient lot.

	Hepatocellular carcinoma		Liver metastases	
	Men	Women	Men	Women
Number of cases	17	4	20	8
Median age (min/max)∗	54 (48/69)	59 (44/68)	51 (43/66)	50 (46/70)

Preexisting conditions				
Chronic viral hepatitis B	3	0	0	0
Chronic viral hepatitis C	2	1	1	0
Cirrhosis (B)	6	3	0	0
Cirrhosis (C)	3	0	0	0
Cirrhosis (B and C)	2	0	0	0
Other malignancies	0	0	20	8
Alcohol consumption	9	0	8	0
Smoking	11	2	14	3

Characteristics of the tumor				
Single tumor∗∗	17	4	17	6
Median size (min/max)∗∗∗	1.9 (1.0/2.0)	1.7 (1.0/1.9)	6.1 (2.4/7.1)	5.9 (2.1/8.2)

*Age in years; ∗∗for multiple tumors, only the largest in size is reported in the table; ∗∗∗diameter in centimeters.

**Table 2:** Number of correct interpretations of random images by the first pathologist.

Identified as…	Correct diagnosis		
	HCC	LM	Normal tissue
HCC	1046	4	0
LM	22	1378	0
Normal tissue	0	0	2450

**Table 3:** Number of correct interpretations of random images by the second pathologist.

Identified as…	Correct diagnosis		
	HCC	LM	Normal tissue
HCC	1044	6	0
LM	17	1383	0
Normal tissue	0	0	2450

**Table 4:** Distribution of FDs obtained for individual cell nuclei and blood vessels via automated analysis. This data constituted input parameters for the first ANN.

	Cell nuclei	Blood vessels
Median FD per element		
HCC	1.78	1.83
LM	1.64	1.41
Normal tissue	1.21	1.12
Minimum FD per element		
HCC	1.23	1.63
LM	1.18	1.11
Normal tissue	1.03	1.02
Maximum FD per element		
HCC	1.91	1.96
LM	1.94	1.63
Normal tissue	1.68	1.36

**Table 5:** Number of correct interpretations of random images (after the completion of the training phase) by the ANN system.

Identified as…	Correct diagnosis		
	HCC	LM	Normal tissue
HCC	947	103	0
LM	185	1215	0
Normal tissue	27	30	2403

**Table 6:** The level of agreement between the CAD system relying on FDs and the human operators that subjectively evaluated the images.

Comparison	Kappa	Standard error	95% confidence interval	Force of concordance
Observer 1 and CAD	0.978	0.003	0.973–0.983	Excellent
Observer 2 and CAD	0.898	0.005	0.887–0.909	Excellent
